# Genomic Analysis of a New Freshwater Cyanophage Lbo240-yong1 Suggests a New Taxonomic Family of Bacteriophages

**DOI:** 10.3390/v15040831

**Published:** 2023-03-24

**Authors:** Qin Zhou, Dengfeng Li, Wei Lin, Linting Pan, Minhua Qian, Fei Wang, Ruqian Cai, Chenxin Qu, Yigang Tong

**Affiliations:** 1 Key Laboratory of Marine Biotechnology of Zhejiang Province, School of Marine Sciences, Ningbo University, Ningbo 315211, China; 2 College of Life Science and Technology, Beijing University of Chemical Technology, Beijing 100029, China

**Keywords:** cyanophage, isolation, genome, *Leptolyngbya boryana*, phylogenetic tree

## Abstract

A worldwide ecological issue, cyanobacterial blooms in marine and freshwater have caused enormous losses in both the economy and the environment. Virulent cyanophages—specifically, infecting and lysing cyanobacteria—are key ecological factors involved in limiting the overall extent of the population development of cyanobacteria. Over the past three decades, reports have mainly focused on marine *Prochlorococcus* and *Synechococcus* cyanophages, while information on freshwater cyanophages remained largely unknown. In this study, a novel freshwater cyanophage, named Lbo240-yong1, was isolated via the double-layer agar plate method using *Leptolyngbya boryana* FACHB-240 as a host. Transmission electron microscopy observation illustrated the icosahedral head (50 ± 5 nm in diameter) and short tail (20 ± 5 nm in length) of Lbo240-yong1. Experimental infection against 37 cyanobacterial strains revealed that host-strain-specific Lbo240-yong1 could only lyse FACHB-240. The complete genome of Lbo240-yong1 is a double-stranded DNA of 39,740 bp with a G+C content of 51.99%, and it harbors 44 predicted open reading frames (ORFs). A Lbo240-yong1 ORF shared the highest identity with a gene of a filamentous cyanobacterium, hinting at a gene exchange between the cyanophage and cyanobacteria. A BLASTn search illustrated that Lbo240-yong1 had the highest sequence similarity with the *Phormidium* cyanophage Pf-WMP4 (89.67% identity, 84% query coverage). In the proteomic tree based on genome-wide sequence similarities, Lbo240-yong1, three *Phormidium* cyanophages (Pf-WMP4, Pf-WMP3, and PP), one *Anabaena* phage (A-4L), and one unclassified *Arthronema* cyanophage (Aa-TR020) formed a monophyletic group that was more deeply diverging than several other families. Pf-WMP4 is the only member of the independent genus *Wumpquatrovirus* that belongs to the *Caudovircetes* class. Pf-WMP3 and PP formed the independent genus *Wumptrevirus. Anabaena* phage A-4L is the only member of the independent *Kozyakovvirus* genus. The six cyanopodoviruses share similar gene arrangements. Eight core genes were found in them. We propose, here, to set up a new taxonomic family comprising the six freshwater cyanopodoviruses infecting filamentous cyanobacteria. This study enriched the field’s knowledge of freshwater cyanophages.

## 1. Introduction

With the rapid development of industry and agriculture in recent decades, serious anthropogenic eutrophication and mass developments of cyanobacteria in water, called “water blooms” or “cyanobacterial blooms”, have become a common occurrence worldwide [1,2,3]. Water blooms are frequently associated with cyanotoxins, posing a health hazard to other aquatic organisms and drinking waters [4,5]. Planktonic viruses, especially cyanobacterial viruses (cyanophage), are important aquatic ecological factors involving the regulation of the population of plankton, especially cyanobacteria. Up to 20% of prokaryotic organisms in the oceans are estimated to die every day due to viral infection and lysis [6]. Viruses, therefore, play a key role in the biogeochemical cycling and host mortality, metabolism, physiology, and evolution in the ocean that are driven by these organisms [7,8].

Cyanophages are viruses that infect and lyse cyanobacterial cells [8,9]. Cyanophage infection and lysis are closely related to the control of the reproduction and termination of toxic cyanobacteria [10]. Cyanophages have become a research hotspot. In the past, studies mainly focused on marine cyanophages, especially *Prochlorococcus* and *Synechococcus* cyanobacteria [11]. Little information about freshwater cyanophages has been obtained. Retrieval of the existing literature and GenBank databases revealed that the coding densities of freshwater cyanophage genomes were 89%–94%, and freshwater cyanophages usually lack homologs of photosynthetic genes prevalent in marine cyanophages [12,13,14]. To date, only ten freshwater cyanophages infecting *Leptolyngbya boryana* (formerly *Plectonema boryanum*) were reported (Table 1). Of those, only two (PP and MinS1) were sequenced and characterized [13,14].

Here, we isolated a new lytic *Leptolyngbya* cyanophage, Lbo240-yong1, from freshwater using *L.boryana* FACHB-240 as the indicator host. The *Podovirus-like* cyanophage Lbo240-yong1 has a narrow host range. Its genome was sequenced and analyzed, and 92.05% of the genome was predicted to be comprised of coding sequences. Bioinformatics analysis suggested that it is justified to create a new taxonomic family comprising six *Podovirus-like* cyanophages infecting filamentous freshwater cyanobacteria, which share at least eight core genes (DNA polymerase, DNA primase/helicase, capsid protein, tail protein, tail tubular protein A, tail tubular protein B, murein hydrolase activator, and terminase large subunit).

## 2. Materials and Methods

### 2.1. Isolation of Cyanophages

The surface water sample was collected from Lake Sunhu (North latitude, 29.982345; East longitude, 121.502455) of Ningbo, Zhejiang province, People’s Republic of China, on 20 November 2021. The water sample was centrifuged (12,000× *g*, 15 min, 4 °C). The supernatant was filtered successively through medium-speed filter paper and 0.45 µm and 0.22 µm polyethersulfone filters (ANPEL Laboratory Technologies, Shanghai, China; product no.14541871). Then, 30 mL of the filtered supernatant of the water sample, 12 mL of a 5 × BG11 medium, and 30 mL of *L. boryana* FACHB-240 (OD_680_ ≈ 0.6) were mixed in a conical flask. The mixtures were cultured in a light incubator under a light: dark cycle of 12: 12 h with a constant illumination of 40 µmol photons m^−2^ s^−1^ at 25 °C. The yellowed culture was centrifuged (8000× *g*, 20 min, 4 °C). The supernatant was co-cultured with fresh *L.boryanum* FACHB-240 again. The above steps were repeated three times.

The double-layer agar method was used to isolate cyanophages with a little modification [22,23]. Lysates were centrifuged (8000× *g*, 20 min, 4 °C), filtered through 0.45 µm and 0.22 µm polyethersulfone filters, and diluted with BG11 (10^−1^–10^−9^). Each 200 µL of dilution was mixed with 1.8 mL of concentrated (1:15) FACHB-240 cultures during the logarithmic phase, incubated in a light incubator for 30 min, and then mixed rapidly with 10 mL of molten BG11 agar medium (0.7% agar, pre-incubated at 42 °C), spread onto BG11 agar plates (1.5% agar). Clarified plaques appeared within 3–7 days. A unique plaque was picked, suspended in 1 mL of BG11 medium, and subsequently used for a new round of plaque isolation. The plaque assay was repeated 5 times until plaques of uniform shape and size were obtained. A single plaque of the 5th generation was picked and suspended in 3 mL of FACHB-240 cultures during the logarithmic phase for 1 day. The lysate was centrifuged at 10,000× *g* for 10 min at 4 °C, and the supernatant was filtered through 0.22 µm polyethersulfone filters. The amplification culture was developed through the co-cultivation of *L. boryana* FACHB-240 and the filtrate at a volume ratio of 8:1 until the culture turned yellow.

### 2.2. Preparation of Cyanophage Suspensions

Lysates were centrifuged (8000× *g*, 20 min, 4 °C), and the supernatants were filtered through 0.45 µm and 0.22 µm polyethersulfone filters. The filtrates were layered on top of the sucrose density gradients (20–40%) and then centrifuged (40,000× *g*, 1 h, 4 °C). The pellets were suspended in 0.01 M of PBS at 1/10 of the original volume of the lysates and dialysed against PBS at 4 °C. Then, BG11 medium was added to make the final volume of the cyanophage suspension equal to reach the original volume of the lysates.

### 2.3. Electron Microscopy Observation

Lbo240-yong1 suspensions were deposited on 400 mesh copper grids for 10 min and negatively stained for 30 s with 2% uranyl acetate (Sigma-Aldrich, St. Louis, MO, USA). Photographs were taken under transmission electron microscopy (Hitachi-7650, Tokyo, Japan) with a magnification of 80,000× at 60 kV. *L. boryana* FACHB-240 cultures infected with Lbo240-yong1 were negatively stained and observed in a similar way.

### 2.4. Host Range Experiments

Thirty-seven cyanobacterial strains (Table S1) were obtained from the Freshwater Algae Culture Collection at the Institute of Hydrobiology (FACHB), Academy of Sciences, Wuhan, China. The phage suspension was mixed with the cyanobacterial cultures during the exponential growth phase at a volume ratio of 1:2 in 48-well cell culture plates, and incubated in a light incubator under a light:dark cycle of 12: 12 h with a constant illumination of 40 µmol photons m^−2^ s^−1^ at 25 °C. In the control groups, phage suspension was replaced with BG11. All cultures were monitored daily for the liquid color, density, integrity, transparency, and boundary clarity of the cells via visual inspection, optical microscopy observation, and OD_680_ measurement. Cyanobacterial strains that did not lyse until the 15th day were defined as unsusceptible.

### 2.5. DNA Isolation, Genome Sequencing

Lysates were centrifuged (8000× *g*, 20 min, 4 °C) and filtered through 0.45 µm and 0.22 µm polyethersulfone filters. Genomic DNA was extracted with the High Pure Viral RNA kit (Roche, Basel, Switzerland, product no. 11858882001), which allows for the extraction of DNA and RNA together. A 2 × 300 bp paired-end DNA library was constructed using the NEBNext Ultra™ II DNA Library PrepKit for Illumina. Genome sequencing of the cyanophage was performed using the Illumina MiSeq (San Diego, CA, USA) sequencing platform to obtain paired-end reads. The control group of FACHB-240 cultures without cyanophages was sequenced in the same way. For the raw sequencing data, the reads present in the control group were deleted from the experimental group. Then, the low-quality (Q-value <20) reads and adapters were filtered out using Trimmomatic-0.36. The clean reads were assembled using SPAdes 3.13.0 software (http://cab.spbu.ru/software/spades/; accessed on 18 March 2022). Genome termini were predicted both by using the proposed method [24] and using PhageTerm online (https://sourceforge.net/projects/phageterm; accessed on 18 March 2022) [25]. The CRISPR/Cas and CRISPR spacer were analyzed using the CRISPRs web server (https://crispr.i2bc.paris-saclay.fr/; accessed on 18 March 2022) [26,27] and Integrated Microbial Genome/Virus (IMG/VR) version 3 (https://img.jgi.doe.gov/; accessed on 18 March 2022) (*E*-value < 10^−^^5^) [28]. The tRNA gene was predicted by using tRNAs-can-SE software (http://lowelab.ucsc.edu/tRNAscan-SE/) [29].

### 2.6. Genome Annotation

Gene prediction was initially executed with the Rapid Annotation using Subsystem Technology (RAST) annotation server (http://rast.nmpdr.org/; accessed on 18 March 2022) [30]. All predicted open reading frames (ORFs) were verified by searching against the nr database with BLASTp (*E*-value ≤ 10^−5^), all of the databases linked with Hmmer (Pfam, TIGRFAM, Gen3D, Superfamily, PIRSF, and Treefam) (https://www.ebi.ac.uk/Tools/hmmer/search/hmmscan; accessed on 18 March 2022) (*E*-value ≤ 10^−5^), and the linked databases of HHpred (https://toolkit.tuebingen.mpg.de/#/tools/hhpred; accessed on 18 March 2022) [31] (*E*-value ≤ 10^−5^, percentage possibility of homologous sequences >96%).

### 2.7. Taxonomic Analysis

Initial genome comparisons were conducted using the BLASTn server of NCBI (*E*-value ≤ 10^−5^) with the Lbo240-yong1 genome (18 March 2022). The average nucleotide identity (ANI) value between Lbo240-yong1 and the closest cyanophage, Pf-WMP4 (NC_008367.1), based on BlastN comparison, was calculated using the EzGenome web server [32]. The in silico DNA–DNA hybridization (isDDH) identity between the Lbo240-yong1 and Pf-WMP4 was calculated using the GGDC web server [33]. The Pairwise Sequence Comparison (PASC) tool (https://www.ncbi.nlm.nih.gov/sutils/pasc/viridty.cgi) [34] and VIRIDIC (http://rhea.icbm.uni-oldenburg.de/VIRIDIC/) [35], respectively, were used to calculate the nucleotide sequence similarity and nucleotide-based intergenomic similarity between Lbo240-yong1 and all phages in the current versions of the databases (18 March 2022). ViPTree online [36] (https://www.genome.jp/viptree/) (accessed on 18 March 2022) was used to generate a proteomic tree based on genome-wide sequence similarities. Computed using tBLASTx, for Lbo240-yong1, all seven *Podovirus-like* freshwater cyanophages were reported with genomes: one *Siphoviridae*-like freshwater cyanophage that can infect *L. boryana,* five *Podovirus-like* marine cyanophages [37,38,39], and 59 phage species of 33 families of the class *Caudoviricetes*. VirClust [40] (https://rhea.icbm.unioldenburg.de/shiny/ VirClust18/) (accessed on 1 November 2022) was used for virus clustering and core protein analysis. Maximum likelihood phylogenetic trees were constructed using MEGA X [41] (5 November 2022) based on the core proteins of six related freshwater cyanopodoviridae and on the terminase large subunit of six related freshwater cyanopodoviruses and outgroups. Four freshwater metagenomes (PRJNA846077, PRJNA869295, PRJNA497963, and PRJNA848245) were downloaded from genbank. The metagenomic reads were assembled using MEGAHIT (v1.1.1) sofware [42] (18 March 2022). The assembled contigs, 20 ~ 200 kb in length, were assessed using VirSorter2 software to identify and select the phage-related ones [43]. VirSorter2 is a multi-classifier, expert-guided approach for detecting diverse viruses [43]. A gene-sharing network analysis of the contigs and Lbo240-yong1 was performed using vConTACT2 (v0.9.13) (10 November 2022), as vConTACT2 is a useful tool for examining distance measures between pairs of genomes and offers a scalable, robust, systematic, and automated means through which to classify virus sequences [44].

## 3. Results

### 3.1. Cyanophage Isolation

**Figure 1:**
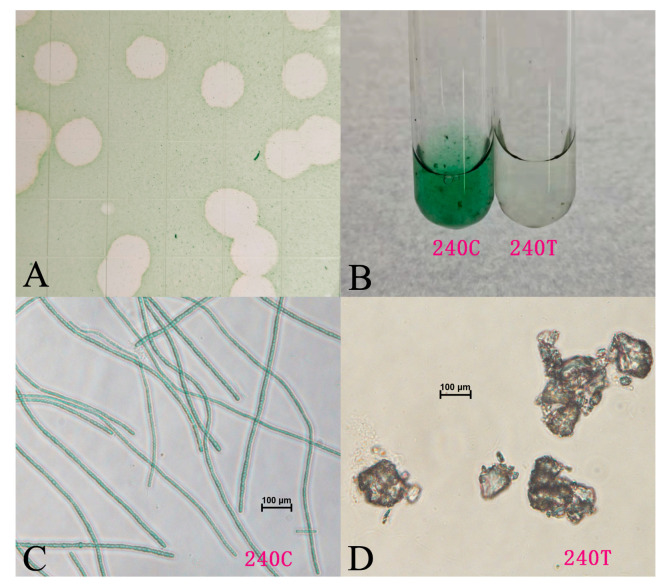
Normal and microscopic pictures of *L. boryana* FACHB-240 cultures: (**A**) plaques developed by Lbo240-yong1 on the FACHB-240 lawn; (**B**) picture of a FACHB-240 culture (left) and of a FACHB-240 culture infected with Lbo240-yong1 (right); (**C**) microscopic picture of a normal FACHB-240 culture; (**D**) microscopic picture of a FACHB-240 culture infected with Lbo240-yong1.

The experimental *L. boryana* FACHB-240 cultures became yellow 18 h after the addition of the filtrate of the water sample collected from Sun Lake. Soon afterwards, the cultures turned colorless and transparent. Contemporaneously, the control groups remained turbid and blue-green. After five successive single-plaque isolations, Lbo240-yong1 developed uniformly big, round, and clear plaques without halos in 3 days (Figure 1A). A single plaque of the 5th generation made the logarithmic FACHB-240 cultures colorless within 1 day (Figure 1B). The algal filament of the infected L.boryanum FACHB-240 fractured, and the cells gathered into masses and then died, leading to a sharp decline in the density of living cells (Figure 1C,D).

### 3.2. General Features of Lbo240-yong1

**Figure 2:**
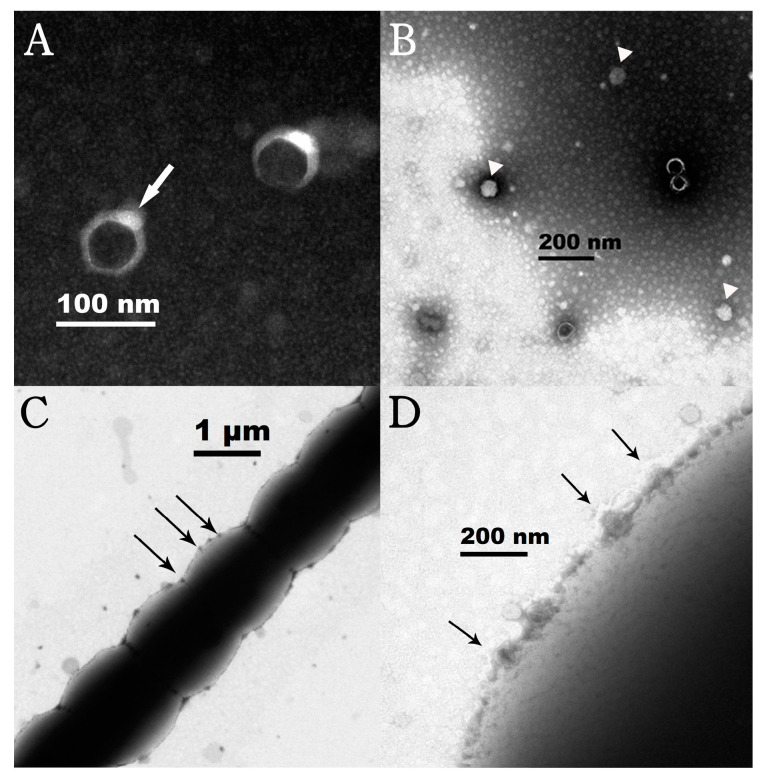
Transmission electron microscopy images of negatively stained free Lbo240-yong1 (**A**,**B**) and *L. boryana* FACHB-240 cells absorbed by Lbo240-yong1 (**C**,**D**). Lbo240-yong1 had an icosahedral head of 50 ± 5 nm in diameter and a short tail of 20 ± 5 nm in length. The white arrow indicates the negatively stained Lbo240-yong1. Black Arrows indicate a large number of cyanophage particles, adsorbed on the surfaces of host cells. White triangles indicate particles are intact and full of DNA.

TEM observation revealed that the negatively stained Lbo240-yong1 had an icosahedral head of 50 ± 5 nm in diameter and a short tail when viewed at the correct angle (Figure 2A) (white arrow). It is morphologically similar to the *Phormidium* cyanophage Pf-WMP4 [45], which possesses an icosahedron head (about 55 nm in diameter) attached to a short tail. The white particles (arrow head) are intact; the particles with black heads in the center may be depleted of DNA (Figure 2B). Under electron microscopy, a large number of cyanophage particles (black arrow), adsorbed on the surfaces of host cells, were observed (Figure 2C,D).

In the host range experiments, 37 cyanobacterial strains (Table S1) were co-cultured with Lbo240-yong1 in triplicate. The results showed that only the indicator host, *L. boryana* FACHB-240, was susceptible to Lbo240-yong1.

### 3.3. Genomic Analysis of Lbo240-yong1

The complete genome sequence of Lbo240-yong1 was sequenced, with an average sequencing depth of 89-fold, using next-generation sequencing (NGS). The SPAdes assembly shaped a complete genome with 127 bp kmer located at both ends. Bandage analysis showed that the assembled product was a circular molecule. Except for Lbo240-yong1, no other phage was found in NGS. The Lbo240-yong1 genome was 39,740 bp in length with a G + C content of 51.99%, and 92.05% of the Lbo240-yong1 genome was occupied by coding sequences. No tRNA gene was found in the Lbo240-yong1 genome. Terminal analysis revealed that the genome of Lbo240-yong1 had preferred termini with terminal redundancy. The complete genome sequence of Lbo240-yong1 was deposited in GenBank (https://www.ncbi.nlm.nih.gov/genbank, accessed on 30 March 2022) under the accession number OM897575.

The Lbo240-yong1 genome contained 44 predicted open reading frames (ORFs) which encoded proteins/peptides of 40 to 1544 AA residues in length. Of all the 44 ORFs, 39 (88.6%) had an ATG initiation codon, 3 (6.8%) had a GTG initiation codon, and 2 (4.6%) had a TTG initiation codon. The best hits of BLASTp scanning with the 44 predicted ORFs of Lbo240-yong1 are summarized in Table 2. In total, 35 ORFs shared the highest identity with Pf-WMP4 genes; 8 ORFs had no BLASTp hit. Interestingly, one ORF (ORF5) of Lbo240-yong1 shared the highest identity with a gene (encoding a Gp49 family protein) of a filamentous cyanobacterium (Calothrix sp. strainPCC 7716), which hints at a gene exchange between cyanophage Lbo240-yong1 and cyanobacteria. No ORF was found to be associated with virulence factors and antibiotic resistance genes, which is advantageous for the application development of the cyanophage.

Three matching sequence fragments between the Lbo240-yong1 genome and viral spacer sequences were found within the CRISPRs databases (*E*-value < 10^−5^). The three sequences were as follows: CCACCCACACGGGGGGACGGGCGCGCCACCTATATGTA (nt 39095-39132), AGAGAATTCCATCTGCATCAATGCACCCCCCATATTCTA (nt 34313-34351), and ATAATCTGGGGGGTAAGCTGCGCTAGCAGCAGCATCCGGTG (nt 30331-30371). These fragments share similarities with the viral spacers of *Leptolyngbya* sp. FACHB-161 (Bit Score, 64.4; *E*-value, 10^−^^8^; identity, 97%), *Leptolyngbya* sp. FACHB-239 (Bit Score, 57.2; *E*-value, 10^−^^6^; identity, 92%), *L. boryana* FACHB-402 (Bit Score, 57.2; *E*-value, 10^−^^6^; identity, 90%), *Leptolyngbya* sp. FACHB-238 (Bit Score, 57.2; *E*-value, 10^−^^6^; identity, 90%), *L. boryana* IAMM-101 (Bit Score, 64.4; *E*-value, 10^−^^8^; identity, 97%), *L. boryana* PCC 6306 (Bit Score, 57.2; *E*-value, 10^−^^6^; identity, 92%), *L. boryana* dg5 (Bit Score, 57.2; *E*-value, 10^−^^6^; identity, 90%), and *L. boryana* NIES-2135 (Bit Score, 64.4; *E*-value, 10^−^^8^; identity, 97%).

**Figure 3:**
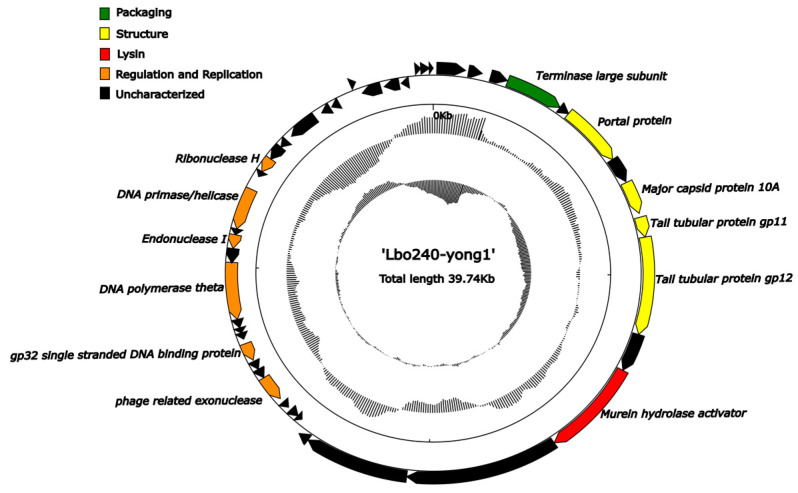
Genome map of cyanophage Lbo240-yong1. The outermost circle represents the 44 ORFs encoded in the genome, with different colors representing different functions (the clockwise arrow indicates the forward reading frame, and the counterclockwise arrow indicates the reverse reading frame); the dark circles in the middle represent the GC content (outwards indicates greater than the average GC content compared with the whole genome, and inwards indicates the opposite); the innermost circle represents the GC skew (G-C/G+C; outwards indicates >0, and inwards indicates <0).

The circular genome map is shown in Figure 3. The predicted Lbo240-yong1 ORFs could be classified into five functional modules: regulation and replication (6 ORFs), structure (4 ORFs), packaging (1 ORF), lysin (1 ORF), and uncharacterized (32 ORFs).

### 3.4. Taxonomic Analysis

A GenBank BLASTn search with the Lbo240-yong1 genome showed that Lbo240-yong1 shared the highest sequence similarity (89.67% identity, 84% query coverage) with *Phormidium* cyanophage Pf-WMP4 (NC_008367.1). Pf-WMP4, also called *Wumpquatrovirus* WMP4—being the only member of the independent genus *Wumpquatrovirus* (rank: *Duplodnaviria*› *Heunggongvirae*› *Uroviricota*› *Caudoviricetes*› *Wumpquatrovirus*)—was assigned directly to the class *Caudoviricetes* by the International Committee on Taxonomy of Viruses (ICTV). The isDDH and ANI values between Lbo240-yong1 and Pf-WMP4 were 34.70% and 88.41%, respectively. These values are below the threshold for isDDH (70%) and ANI (95%) to discriminate viral species, thus demonstrating Lbo240-yong1 as a novel cyanophage species. In PASC and VIRIDIC scanning, Lbo240-yong1 shared the highest nucleotide sequence similarity (79.87%) and the highest nucleotide-based intergenomic similarity (79.5%) with Pf-WMP4. Both similarities were higher than the ≥70% threshold required to define a genus, indicating Lbo240-yong1 as the second member of the *Wumpquatrovirus* genus.

**Figure 4:**
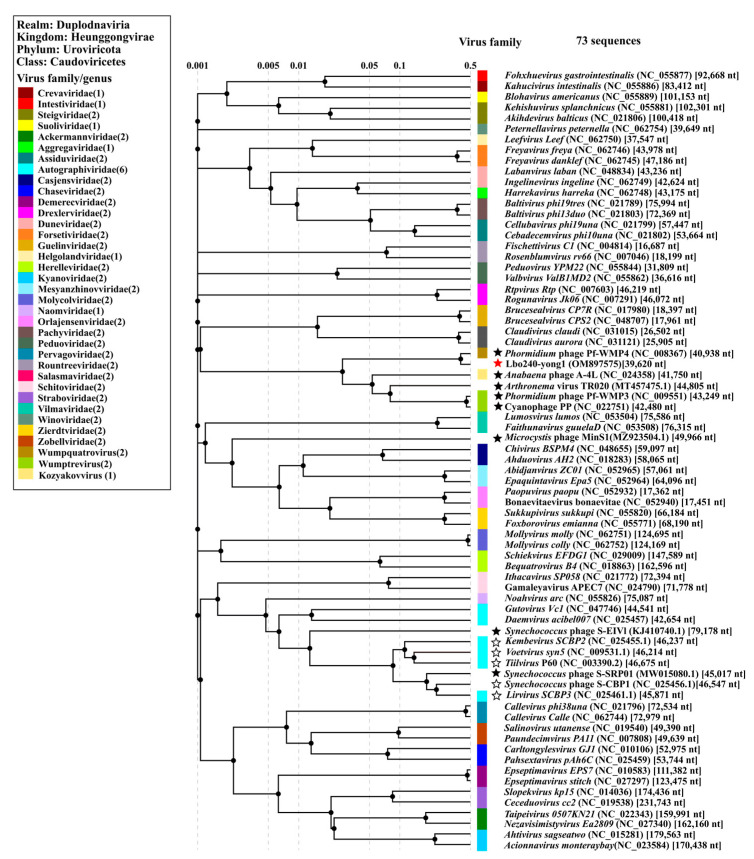
Proteomic tree based on the complete genome sequences of *L. boryana* cyanophage Lbo240-yong1 (red star), all six remaining *Podovirus-like* freshwater cyanophages with reported genomes, one Siphoviridae-like freshwater cyanophage (black star) capable of infecting *L. boryana*, five *Podovirus-like* marine cyanophages (black hollow star), and 59 representative bacteriophages of 33 families of the Caudovirales class. The proteomic tree was generated using ViPTree online, based on the genome-wide similarities determined by tBLASTx. Cyanophage family assignments, according to the official ICTV classification (2022), are provided with different color bars.

In March of 2022, ICTV made a huge update to the phage classification system, which abolished the *Caudovirales* order and three morphologic-based taxa (*Siphoviridae*, *Myoviridae*, and *Podoviridae*) that have been repeatedly shown not to be monophyletic (2021.001B, https://ictv.global/taxonomy/taxondetails?taxnode_id=20171285, accessed on 30 March 2022) [46]. The abolished *Siphoviridae* family once harbored all the phages with long non-contractile tails; the abolished *Myoviridae* family once harbored all the phages with straight contractil tails; the abolished *Podoviridae* family once harbored all the phages with short noncontractile tails. In the updated classification system of the ICTV, *Podovirus-like* phages containing short noncontractile tails were classified in different orders, families, and genera. The order *Caudovirales* was replaced by the class *Caudoviricetes* to group all tailed bacterial and archaeal viruses with icosahedral capsids and a double-stranded DNA genome. The new *Caudoviricetes* class comprises 4 orders (*Crassvirales*, *Kirjokansivirales*, *Methanobavirales*, and *Thumleimavirales*), 33 independent families, 37 independent subfamilies, and 493 independent genera directly to the class *Caudoviricetes* [46]. The new taxonomy release (#37) can be found on the ICTV website (https://ictv.global/, accessed on 30 March 2022). A proteomic tree, based on the genome-wide sequence similarities of 73 reference sequences (including all 12 reported *Podovirus-like* cyanophages with sequenced genomes and all 3 sequenced cyanophages capable of infecting *Leptolyngbya* cyanobacteria (Figure 4), was established. In the tree, Lbo240-yong1 and five freshwater cyanopodoviruses form a monophyletic group that is more deeply diverging than other families. The evolutionary distances between this family and all other sequences were maximal. We propose the creation of a novel family, *Filumcyanopodoviridae*, within the class *Caudoviricetes*, that comprises the six freshwater cyanopodoviruses (*Leptolyngbya* phage Lbo240-yong1; *Phormidium* phages Pf-WMP4, Pf-WMP3, PP; *Arthronema* phage Aa-TR020; and *Anabaena* phage A-4L) infecting filamentous freshwater cyanobacteria [47,48,49]. In the proposed new family, *Leptolyngbya* cyanophage Lbo240-yong1 and *Phormidium* cyanophage Pf-WMP4 belong to the *Wumpquatrovirus* genus, *Phormidium* cyanophage Pf-WMP3 and PP form the *Wumptrevirus* genus, and *Anabaena* phage A-4L represents the *Kozyakovvirus* genus, while *Arthronema* cyanophage Aa-TR020 has not been classified. These freshwater cyanopodoviruses, except for the lysogenic cyanophage Aa-TR020, are lytic. Except for Lbo240-yong1, the five cyanophage genomes all contain long terminal repeats (107–234 bp). The absence of the genome terminal repeats of Lbo240-yong1 may be due to recombination, the jumping of mobile elements, or other factors. The genome sizes of the six cyanophages are approx. 40 kb to 45 kb (Table 3). In total, there are seven *Podovirus-like* freshwater cyanophages with sequenced genomes. The proposed new family harbors six of them. The exception is *Synechococcus* cyanophage S-SRP01, which was reported to have a high degree of similarity with marine cyanophages [50]. In the proteomic tree (Figure 4), S-SRP01 and two marine *Synechococcus* cyanopodoviruses form a monophyletic clade.

**Figure 5:**
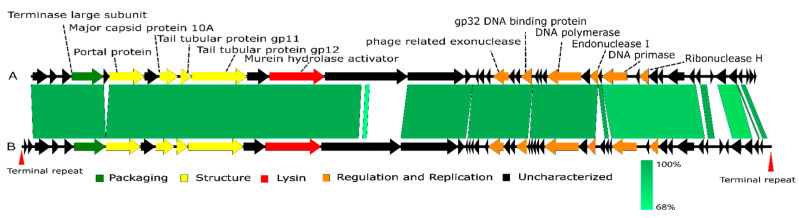
Genome comparison of the Lbo240-yong1 (**A**) and *Phormidium* cyanophage Pf-WMP4 (**B**). The color of each arrow refers to the functional groups. The orientation of the arrows indicates the direction of gene transcription. The homologous regions are represented by gray bars, with their depth reflecting the degree of sequence similarity.

A genome comparison of Lbo240-yong1 and the closest relative, *Phormidium* cyanophage Pf-WMP4, is shown in Figure 5. Lbo240-yong1 shares 35 homologous ORFs with *Phormidium* cyanophage Pf-WMP4. The arrangements and orientations of these homologous ORFs are essentially the same. At the proteomic level, high sequence identity existed between their terminase large subunit (~95%) and structural proteins, such as major capsid protein (~98%), tail tubular protein (~98%), and portal protein (~98%). Conversely, ORF2, ORF16, ORF23, ORF24, ORF35, ORF37, ORF38, ORF39, ORF41, and ORF44 of Lbo240-yong1 shared low identity (<70%) with Pf-WMP4. The other dissimilar sequences were located at the C-terminal coding region of a large hypothetical protein (ORF 12) in the middle of the genome, next to the ORF predicted to encode a putative murein hydrolase activator.

## 4. Discussion

**Figure 6:**
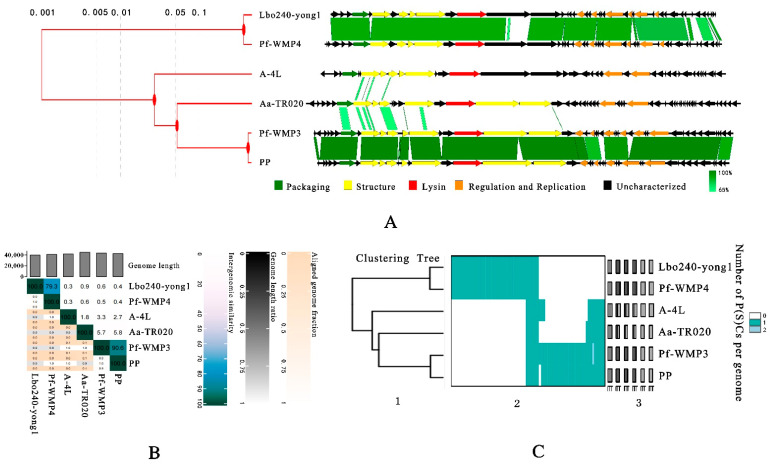
(**A**) Concatenated phylogenetic tree byViPTree (left) and by Easyfig of the six freshwater cyanopodoviruses. The homologous regions are represented by green bars. Light green to dark green represent low to high similarity. (**B**) Percent intergenomic similarities between the six freshwater cyanopodoviruses, calculated by the VIRIDIC server. (**C**) Integrated visualization of the viral clustering output of VirClust. 1. Hierarchical tree calculated using the protein cluster-based intergenomic distances. 2. Heatmap representation of the protein cluster distribution in the viral genomes. Rows represent the individual viral genomes. Columns represent the individual protein clusters. 3. Viral genome-specific statistics: genome length, the proportion of proteins shared (dark grey) from all proteins (light grey bar), and the proportion of proteins shared in the own viral genome cluster.

In the present work, a novel freshwater cyanophage Lbo240-yong1 was isolated. In the phylogenetic tree, Lbo240-yong1 and five cyanopodoviruses infecting filamentous freshwater cyanobacteria formed a monophyletic group. We propose setting up a new family within the class *Caudoviricetes* comprising the six cyanopodoviruses (Lbo240-yong1, Pf-WMP4, Pf-WMP3, PP, A-4L, and Aa-TR020) infecting filamentous freshwater cyanobacteria. Lbo240-yong1, Pf-WMP4, Pf-WMP3, PP, A-4L, and Aa-TR020 share common features (Table 3). Their hosts are all filamentous freshwater cyanobacteria. They are similar in morphology and size, all being *Podovirus-like*, having icosahedral heads (50 nm to 55 nm in diameter) and short tails. Their genomes are similar in size (40 kb to 45 kb), architecture, and gene content (Table 3 and Figure 6A). The genes (associated with phage packaging, structure, and bacteriolysis) located in the first half of the genome of Lbo240-yong1 were predicted to transcribe in the same orientation, while the other genes (associated with replication and regulation) located in the remaining half of the genome were predicted to transcribe in the opposite direction. Similar gene arrangements exist in the other five freshwater cyanopodoviruses (Figure 6A).

The six freshwater cyanopodoviruses of the proposed novel family were distinct from other classified phages. The proposed novel family shares a node with the clade composed of Guelinviridae and Salasmaviridae, at a rudimentary level of similarity of about 0.001 in the proteomic tree, based on genome-wide analysis (Figure 4). The viruses of the Guelinviridae and Salasmaviridae families (Table S2, Figures S1 and S2) contained genomes much smaller than the six cyanopodoviruses (Figure S3) [51,52].

Except for A-4L, five of the six cyanopodoviruses are all host-specific. Seven different cyanobacterial strains were used for the cross-infectivity studies of Pf-WMP4, but only the indicator host, *P. foveolarum* Gom, was susceptible [45]. The host range studies of Pf-WMP3 were tested against 11 cyanobacterial species; only *P. foveolarum* and *P. tenue* were susceptible [47]. PP was reported to infect seven filamentous cyanobacterial strains of two species: *L. boryana* (IU594, FACHB-402, FACHB-246, and FACHB-240) and *P. foveolarum* (FACHB-238, FACHB-239, and FACHB-161) [53]. Aa-TR020 was reported to be strain-specific, only infecting *Arthronema africanum* strain1980/01 [48]. In this study, the host range experiment of Lbo240-yong1 was tested against 37 cyanobacterial species, lysis only appeared in the filamentous *L. boryana* strain FACHB-240. These results agree with a previous research report indicating that cyanopodoviruses are usually highly host-specific [54].

The inter-genomic similarities between the six freshwater cyanopodoviruses were calculated using the VIRIDIC server. Results showed very high similarity (90.6%) between Pf-WMP3 and PP of the *Wumptrevirus* genus *and* high similarity (79.3%) between Lbo240-yong1 and Pf-WMP4 of *the Wumpquatrovirus* genus (Figure 6B). Otherwise, a low degree of similarity (≦5.8%) was shared by *Wumptrevirus*, *Wumpquatrovirus*, *Kozyakovvirus* (A-4L), and Aa-TR020. The G + C contents of *Wumpquatrovirus* (about 52%), *Kozyakovvirus* (about 43%), *Wumptrevirus*, and Aa-TR020 (about 46%) are diverse.

VirClust was used to group orthologous proteins into protein clusters (PCs). Eight core proteins (DNA polymerase, DNA primase/helicase, capsid protein, tail protein, tail tubular protein A, tail tubular protein B, murein hydrolase activator, and terminase large subunit) were found within the six related cyanopodoviruses using VirClust. The eight hallmark genes of the six cyanopodoviruses were then used to build maximum likelihood phylogenetic trees (Figure S4A). Another maximum likelihood phylogenetic tree, based on the terminase large subunit, was constructed using outgroups, demonstrating that the proposed family clearly forms a monophyletic clade (Figure S4B). All phylogenetic trees, whether based on the whole genome (Figure 4 and Figure 6A,C), the core genes (Figure S4A), or the terminase large subunit, were broadly similar, i.e., the six related cyanopodoviruses clustered in the same way and formed a monophyletic clade.

Information about isolated and sequenced freshwater cyanophage is very limited. To find out whether the cyanophages related to Lbo240-yong1 are prevalent, four freshwater metagenomes were downloaded and assembled, a gene-sharing network was inferred using vContact2 software, and the contigs related to Lbo240-yong1 were predicted usingVirSorter2 software.

The homologues of Lbo240-yong1 genes were found in all four metagenomes (Figure S5). Five viral contigs sharing significant similarities with Lbo240-yong1 were found. Four of them were cyanopodoviruses (Pf-WMP4, A-4L, Pf-WMP3, and PP) having been isolated. The other uncharacterized one (32,719 bp) was from Israel’s freshwater ponds and Lake Kinneret. The contig and the six related cyanopodoviruses were used for proteomic tree construction (Figure S4C). Compared with the six related cyanopodoviruses, the unidentified contig is only distantly related, sharing five core genes (capsid protein, tail protein, tail tubular protein B, murein hydrolase activator, and terminase large subunit) with the other six cyanopodoviruses.

The CRISPR/Cas immune system is a defense strategy against extrinsic nucleic acids, such as the virus genomes of bacteria and archaea [55]. The CRISPR loci generally consist of non-continuous direct repeats separated by short stretches of DNA sequences called spacers, and these were related to *cas* genes [56]. Three sequence fragments of Lbo240-yong1 were found to match with the spacers of the *Leptolyngbya* genus via scanning the viral spacer database of IMG/VR (*E*-value < 10^−^^5^). These sequence fragments match the viral spacers of the *Leptolyngbya* genus, including *L.boryana* FACHB-402 (Bit Score, 57.2; *E*-value, 10^−^^6^; identity, 90%). The results suggest that this *Leptolyngbya* spp. may have once been infected by related phages in the past and has formed immune resistance [57]. In the host range test in this study, two *L. boryana* strains (FACHB-402 and FACHB-240) were tested. As a result, it was shown that FACHB-402 is resistant, while FACHB-240 is the susceptible indicator host. The presence of the matching CRISPR spacersin, FACHB-402, may provide immune protection against infection.

In summary, based on the morphology and sequence characteristics, we propose that the Lbo240-yong1 should be classified as a novel species of the *Wumpquatrovirus* genus in the *Caudoviricetes* class. A novel family was proposed to harbor all the reported freshwater cyanopodoviruses with sequenced genomes—except for S-SRP01, which reveals a high degree of similarity with marine cyanophages. The isolation and genome analysis of Lbo240-yong1 enriches the field’s knowledge of cyanophages and provides basic useful information for further research and application development. Due to the limited information in the freshwater cyanophage database, it is vital to isolate and identify more cyanophages from freshwater environments.

## Figures and Tables

**Table 1:** A full list of reported cyanophages capable of infecting *Leptolyngbya* cyanobacteria.

Cyanophage	Accession Number	Genome Length	Host (Reference)	Morphology
PP	NC_022751	42,480 bp	*L. boryanum, Phoridium foveolarum*	*Podovirus-like*
Lbo240-yong1	OM_897575	39,740 bp	*L*. *boryanum*	*Podovirus-like*
LPP-1	-	-	*L*. *boryanum* [15]	*-*
LPP1-G	-	-	*L*. *boryanum* [16]	*-*
LPP2-SPI	-	-	*L*. *boryanum* [17]	*-*
LPP-3	-	-	*L*. *boryanum* [18]	*-*
LPP-DUN1	-	-	*L. boryanum* [19]	*-*
Cyanophage SPlcts1	-	-	*L. boryanum* [20]	*-*
LPP-2	-	-	*L. boryanum* [21]	*-*
MinS1	MZ923504	49,966 bp	*Microcystis aeruginosa, L. boryana* et al.	*Siphovirus-like*

“-” indicates no information was reported.

**Table 2:** ORF analysis of cyanophage Lbo240-yong1.

ORF	Size (aa)	Prediction Function	Best BLAST Hit ^a^	Identity ^b^ (aa)	*E*-Value ^c^
1	282	hypothetical protein	ref|YP_762673.1| PfWMP4_43 [cyanophage Pf-WMP4]	95% (270/283)	0.0
2	136	hypothetical protein	no hit		
3	170	hypothetical protein	ref|YP_762671.1| PfWMP4_41 [cyanophage Pf-WMP4]	98% (167/170)	5 × 10^−118^
4	581	terminase large subunit	ref|YP_762670.1| PfWMP4_40 [cyanophage Pf-WMP4]	95% (553/581)	0.0
5	105	hypothetical protein	ref|WP_224176119.1| Gp49 family protein [*Calothrix* sp. PCC 7716]	53% (46/86)	2 × 10^−29^
6	641	portal protein	ref|YP_762669.1| PfWMP4_39 [cyanophage Pf-WMP4]	98% (631/641)	0.0
7	263	hypothetical protein	ref|YP_762668.1| PfWMP4_38 [cyanophage Pf-WMP4]	93% (244/263)	1 × 10^−120^
8	341	major capsid protein 10A	ref|YP_762667.1| PfWMP4_37 [cyanophage Pf-WMP4]	98% (333/341)	0.0
9	211	tail tubular protein gp11	ref|YP_762666.1| PfWMP4_36 [cyanophage Pf-WMP4]	98% (206/211)	2 × 10^−151^
10	1012	tail tubular protein gp12	ref|YP_762665.1| PfWMP4_35 [cyanophage Pf-WMP4]	98% (994/1012)	0.0
11	400	hypothetical protein	ref|YP_762664.1| PfWMP4_34 [cyanophage Pf-WMP4]	95% (380/400)	0.0
12	1012	murein hydrolase activator	ref|YP_762663.1| PfWMP4_33 [cyanophage Pf-WMP4]	93%(943/1014)	0.0
13	1519	hypothetical protein	ref|YP_762662.1| PfWMP4_32 [cyanophage Pf-WMP4]	74% (661/891)	0.0
14	1047	hypothetical protein	ref|YP_762661.1| PfWMP4_31 [cyanophage Pf-WMP4]	97% (1016/1047)	0.0
15	110	hypothetical protein	ref|YP_762660.1| PfWMP4_30 [cyanophage Pf-WMP4]	97% (107/110)	8 × 10^−70^
16	57	hypothetical protein	no hit		
17	103	hypothetical protein	ref|YP_762658.1| PfWMP4_28 [cyanophage Pf-WMP4]	86% (78/91)	3 × 10^−49^
18	80	hypothetical protein	ref|YP_762657.1| PfWMP4_27 [cyanophage Pf-WMP4]	98% (58/59)	2 × 10^−33^
19	285	phage-related exonuclease	ref|YP_762655.1| PfWMP4_25 [cyanophage Pf-WMP4]	96% (274/285)	0.0
20	108	hypothetical protein	ref|YP_762654.1| PfWMP4_24 [cyanophage Pf-WMP4]	98% (106/108)	3 × 10^−73^
21	100	hypothetical protein	ref|YP_762653.1| PfWMP4_23 [cyanophage Pf-WMP4]	71% (71/100)	2 × 10^−43^
22	198	gp32 single-stranded DNA-binding protein	ref|YP_762652.1| PfWMP4_22 [cyanophage Pf-WMP4]	92% (168/182)	1 × 10^−122^
23	73	hypothetical protein	no hit		
24	58	hypothetical protein	no hit		
25	95	hypothetical protein	ref|YP_762650.1| PfWMP4_20 [cyanophage Pf-WMP4]	91% (86/95)	1 × 10^−56^
26	630	DNA polymerase theta	ref|YP_762649.1| DNA polymerase [cyanophage Pf-WMP4]	94% (594/630)	0.0
27	160	hypothetical protein	ref|YP_762648.1| PfWMP4_18 [cyanophage Pf-WMP4]	95% (152/160)	4 × 10^−109^
28	152	endonuclease I	ref|YP_762647.1| PfWMP4_17 [cyanophage Pf-WMP4]	94% (142/151)	1 × 10^−89^
29	67	hypothetical protein	ref|YP_762644.1| PfWMP4_14 [cyanophage Pf-WMP4]	88% (59/67)	8 × 10^−38^
30	461	DNA primase/helicase	ref|YP_762642.1| DNA primase/helicase [cyanophage Pf-WMP4]	99% (457/461)	0.0
31	65	hypothetical protein	ref|YP_762641.1| PfWMP4_11 [cyanophage Pf-WMP4]	85% (53/62)	5 × 10^−31^
32	167	ribonuclease H	ref|YP_762640.1| PfWMP4_10 [cyanophage Pf-WMP4]	77% (129/167)	5 × 10^−94^
33	170	hypothetical protein	ref|YP_762639.1| PfWMP4_09 [cyanophage Pf-WMP4]	94% (159/170)	6 × 10^−113^
34	95	hypothetical protein	ref|YP_762638.1| PfWMP4_08 [cyanophage Pf-WMP4]	90% (85/94)	7 × 10^−58^
35	323	hypothetical protein	ref|YP_762637.1| PfWMP4_07 [cyanophage Pf-WMP4]	66% (189/288)	7 × 10^−126^
36	104	hypothetical protein	ref|YP_762636.1| PfWMP4_06 [cyanophage Pf-WMP4]	79% (82/104)	2 × 10^−50^
37	87	hypothetical protein	no hit		
38	59	hypothetical protein	no hit		
39	203	hypothetical protein	ref|YP_762634.1| PfWMP4_04 [cyanophage Pf-WMP4]	51% (103/202)	5 × 10^−58^
40	159	hypothetical protein	ref|YP_762633.1| PfWMP4_03 [cyanophage Pf-WMP4]	93% (148/159)	4 × 10^−91^
41	79	hypothetical protein	no hit		
42	56	hypothetical protein	ref|YP_762675.1| PfWMP4_45 [cyanophage Pf-WMP4]	100% (56/56)	3 × 10^−24^
43	87	hypothetical protein	ref|YP_762674.1| PfWMP4_44 [cyanophage Pf-WMP4]	99% (86/87)	2 × 10^−54^
44	40	hypothetical protein	no hit		

^a^ The most closely related protein and its organism. “no hits” indicates no significant hits detected for a particular amino acid sequence. ^b^ Percent identity for top hits in BLASTp searches. Numbers in parentheses provide the length of each alignment. ^c^ The expected number of hits, based on the database size, by chance, as determined by BLASTp analysis.

**Table 3:** Comparison of basic characteristics of the six relative freshwater cyanopodoviruses.

Cyanophage Name	Head Diameter	Tail Length	Accession Number	Genome Length	G + C Content	Host Range
Lbo240-yong1	50 ± 5 nm	20 ± 5 nm	OM_897575	39,740 bp	51.99%	*L.boryana* FACHB-240
Pf-WMP4	55 nm	N	DQ875742.1	40,938 bp	51.8%	*P. foveolarum* Gom
A-4L	50 nm	N	KF356198	41,750 bp	43.4%	*Anabaena* sp. and *Nostoc* sp.
Aa-TR020	50 nm	N	MT457475.1	44,805 bp	46%	*A. africanum* 1980/01
Pf-WMP3	55 nm	N	NC_009551.1	43,249 bp	46.49%	*P. tenue* and *P. foveolarum*
PP	52 nm	N	NC_022751	42,480 bp	46.41%	*L. boryanum* and *P. foveolarum*

“N” indicates that no report about tail length can be found.

## Data Availability

Not applicable.

## Supplementary Materials

The following supporting information can be downloaded at: https://www.mdpi.com/article/10.3390/v15040831/s1, Figure S1: Genome map of phage *Claudivirus aurora*; Figure S2: Genome map of phage *Claudivirus claudi*; Figure S3: Genome comparison of three podoviruses (*Brucesealvirus CPS2*, *Claudivirus claudi*, Lbo240-yong1); Figure S4: Maximum likelihood phylogenetic trees generated using MEGA X and proteomic tree generated using ViPTree online. (A) Maximum likelihood phylogenetic tree based on eight core genes of the six related freshwater cyanopodoviruses by using MEGA X (B) Maximum likelihood phylogenetic tree based on terminase large subunits of the six relative freshwater cyanopodoviruses and outgroups of four phages. (C) Proteomic tree for Lbo240 yong1 genome and the homologues including an uncharacterized contig and four freshwater cyanopodoviruses (Pf WMP4, A 4L, Pf WMP3, and PP) found in the assembled sequences of the metagenomes and the freshwater cyanopodoviruses Aa-TR020; Figure S5: Result of gene-sharing network analysis of Lbo240-yong1 and four freshwater metagenomes; Table S1: Results of the host range analysis of Lbo240-yong1 against 37 cyanobacterial strains; Table S2: Basic characteristics of four podoviruses of *Guelinviridae* and *Northropvirinae.*
