# Supplementary material for: Genomic Analysis of a New Freshwater Cyanophage Lbo240-yong1 Suggests a New Taxonomic Family of Bacteriophages
Source: Viruses. 2023 Mar 24;15(4):831. doi: 10.3390/v15040831 (PMC10140849; doi:10.3390/v15040831)
Supplement: Supplementary file 1 [file viruses-15-00831-s001.zip › viruses-2288627-supplementary.pdf]

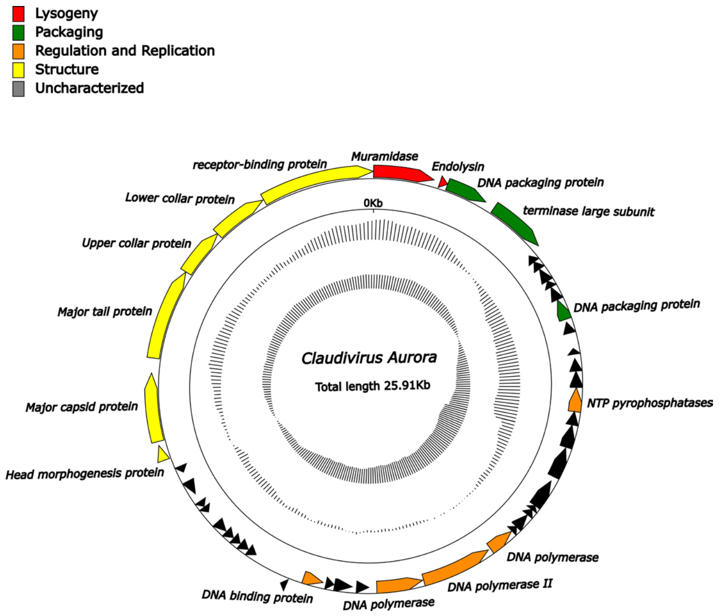

Figure S1. Genome map of phage *Claudivirus aurora.*

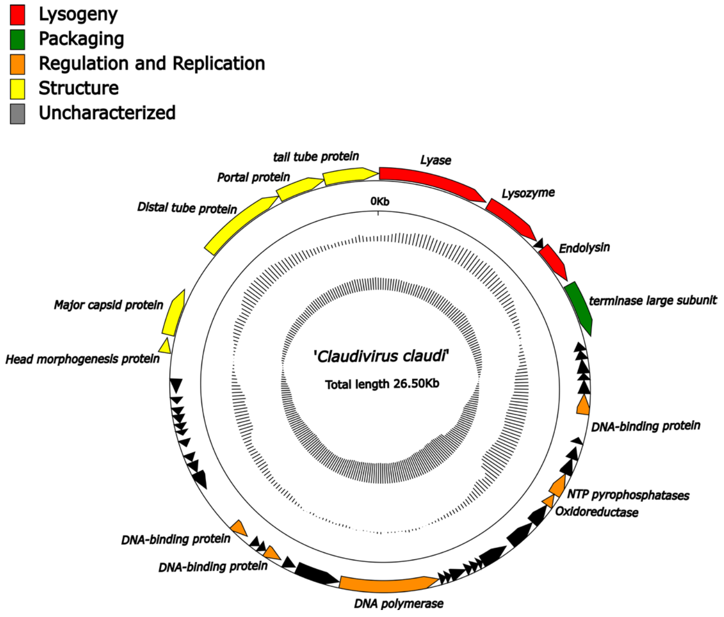

Figure S2. Genome map of phage *Claudivirus claudi.*

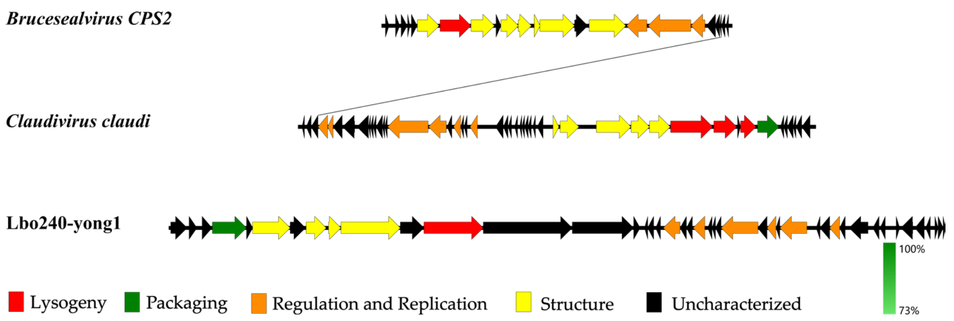

Figure S3. Genome comparison of three podoviruses (*Brucesealvirus CPS2*, *Claudivirus claud*, Lbo240-yong1).

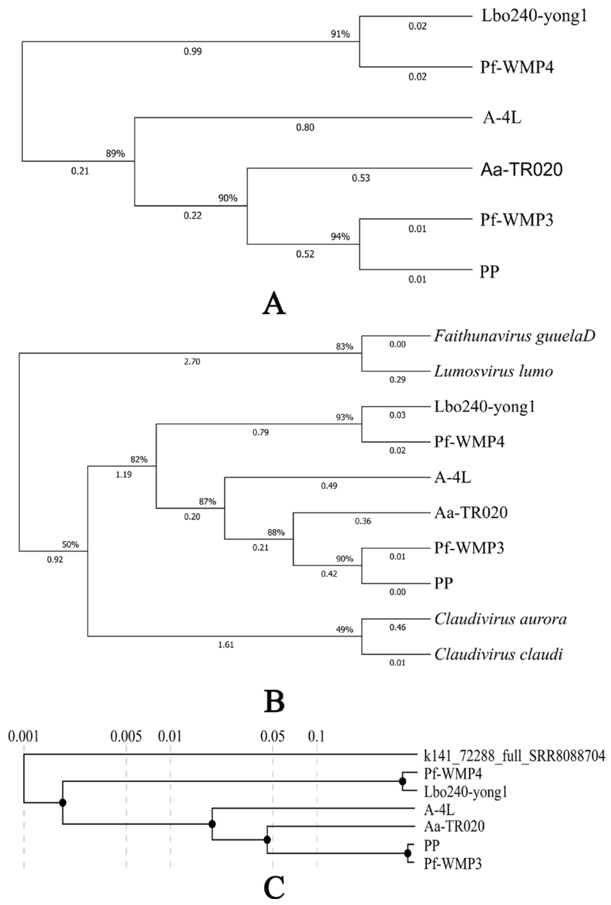

Figure S4 Maximum likelihood phylogenetic trees generated using MEGA X and proteomic tree generated using ViPTree online. (A) Maximum likelihood phylogenetic tree based on eight core genes of the six relative freshwater cyanopodoviruses by using MEGA X. (B) Maximum likelihood phylogenetic tree based on terminase large subunits of the six relative freshwater cyanopodoviruses and outgroups of four phages. (C) Proteomic tree for Lbo240-yong1 genome and the homologues (including an uncharacterized contig and four freshwater cyanopodoviruses: Pf-WMP4, A-4L, Pf-WMP3 and PP) found in the assembled sequences of the metagenomes and the freshwater cyanopodoviruse Aa-TR020.

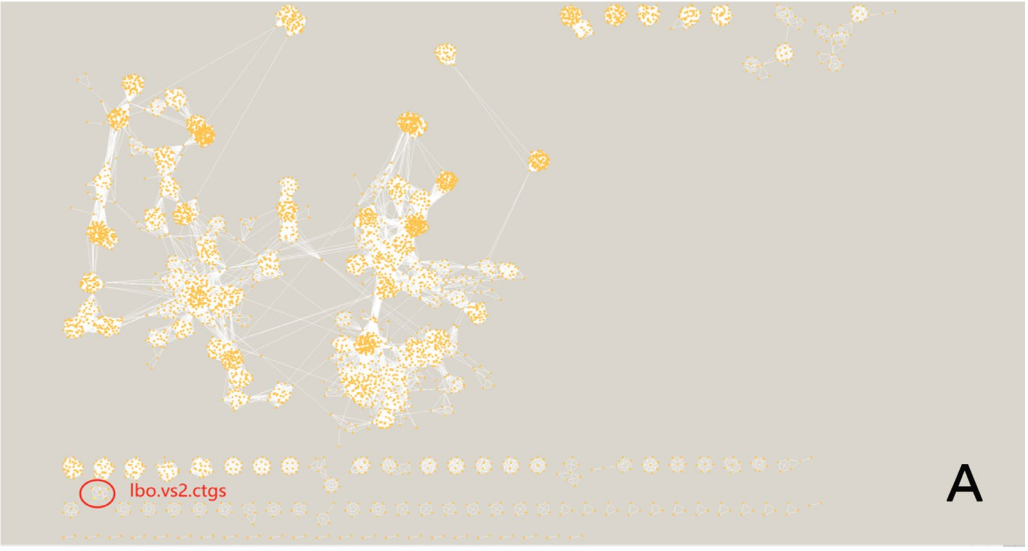

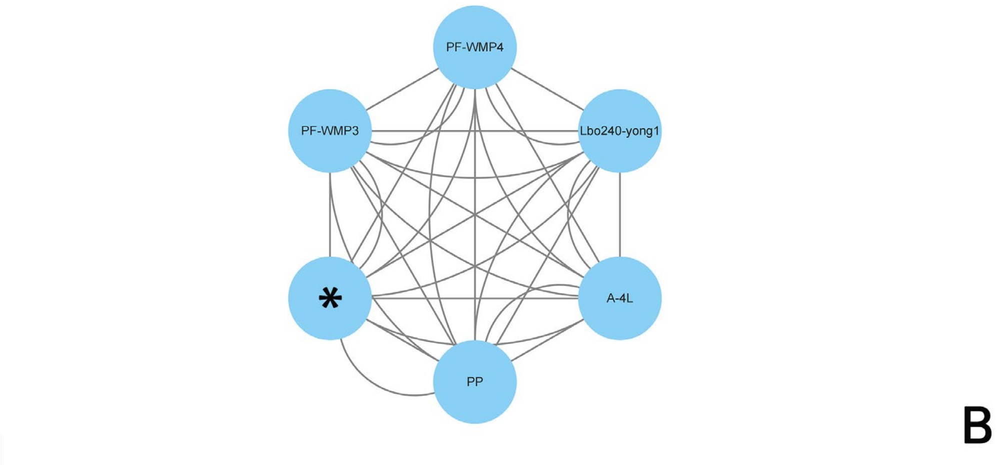

Figure S5. Result of gene-sharing network analysis of Lbo240-yong1 and four freshwater metagenomes.

Table S1. Results of the host range analysis of Lbo240-yong1 against 37 cyanobacterial strains.

Orders	Families	Species	Strains	Susceptibility	Origin
Chroococcales	*Microcystaceae*	*Microcystis aeruginosa*	FACHB-905	-	China
			FACHB-942	-	China
			FACHB-469	-	France
			FACHB-924	-	Australia
			FACHB-925	-	Australia
			FACHB-1326	-	China
		M. wesenbergii	FACHB-929	-	Japan

Orders	Families	Species	Strains	Susceptibility	Origin
			FACHB-1112	-	China
			FACHB-1318	-	China
			FACHB-1317	-	China
		M. flos-aquae	FACHB-1028	-	China
			FACHB-1351	-	China
			FACHB-1323	-	China
		M. elabens	FACHB-916	-	Japan
		M. panniformis	FACHB-1757	-	China
		M. ichthyoblabe	FACHB-1409	-	China
		M. viridis	FACHB-979	-	Japan
			FACHB-1337	-	China
			FACHB-1342	-	China
		Microcystis sp.	FACHB-915	-	France
	Chroococcaceae	*Chroococcus* sp.	FACHB-193	-	China
Nostocales	*Aphanizomenonaceae*	*Aphanizomenon flos-aquae*	FACHB-1039	-	China
			FACHB-1208	-	China
			FACHB-1040	-	China
		Dolichospermum flos-aquae	FACHB-245	-	America
			FACHB-1255	-	China
			FACHB-418	-	France
Oscillatoriales	*Microcoleaceae*	*Planktothrix agardhii*	FACHB-1166	-	China
			FACHB-920	-	Japan
			FACHB-1243	-	China
			FACHB-1261	-	China
		Planktothricoides raciborskii	FACHB-881	-	China
	Oscillatoriaceae	*Oscillatoria planctonica*	FACHB-708	-	China
Synechococcales	*Synechococcaceae*	*Synechococcus* sp.	FACHB-805	-	Australia
		Synechococcus sp.	FACHB-1061	-	China
Pseudanabaenales	*Scytonemataceae*	*Leptolyngbya boryanum*	FACHB-402	-	America
			FACHB-240	+	America

"+" susceptible; "-" not susceptible.

Table S2. Basic characteristics of four podoviruses of *Guelinviridae* and *Northropvirinae.*

Name	Accession number	Head diameter	Tail length	Genome length	G+C content	Morphology	Host range
Brucesealvirus CP7R	NC_017980.1	40–42 nm	35–38 nm	18,397 bp	34.6%	*Podoviridae*	*Clostridium perfringen*
Brucesealvirus CPS2	NC_048707.1	40 nm	15 nm	17,961 bp	33.3%	*Podoviridae*	*C. perfringens*
Claudivirus claudi	NC_031015.2	N	N	26,502bp	30.3%	N	*Bacillus*
Claudivirus aurora	NC_031121.2	N	N	25,905 bp	30.7%	N	*Bacillus*

"N" indicates that no report about tail length can be found.
